# The Attention Training Technique Reduces Anxiety and Depression in Patients With Coronary Heart Disease: A Pilot Feasibility Study

**DOI:** 10.3389/fpsyg.2022.948081

**Published:** 2022-07-27

**Authors:** Toril Dammen, Kristoffer Tunheim, John Munkhaugen, Costas Papageorgiou

**Affiliations:** ^1^Department of Behavioural Medicine, Faculty of Medicine, Insitute of Basic Medical Sciences, University of Oslo, Oslo, Norway; ^2^Division of Mental Health and Addiction, Department of Research and Innovation, Oslo University Hospital, Oslo, Norway; ^3^Faculty of Medicine, Institute of Clinical Medicine, University of Oslo, Oslo, Norway; ^4^Department of Medicine, Drammen Hospital, Drammen, Norway; ^5^Department of Psychology, University of Oslo, Oslo, Norway

**Keywords:** anxiety, depression, coronary heart disease, attention training technique, metacognitive therapy

## Abstract

**Background and Objectives:**

Depression and anxiety symptoms are highly prevalent in coronary heart disease (CHD) patients and associated with poor outcome. Most psychological treatments have shown limited effectiveness on anxiety and depression in these patients. This study evaluates the feasibility of the attention training technique (ATT) in CHD patients with symptoms of anxiety and/or depression.

**Methods:**

Five consecutive CHD patients with significant depression and anxiety symptoms with Hospital Anxiety and Depression rating scale (HADS) -anxiety or -depression subscale score > 8 received 6 weekly group-sessions of ATT in an open trial. Outcomes included feasibility and symptoms measured by HADS, at baseline, post-treatment and at 6 months follow-up. We also assessed psychiatric diagnoses, type D personality, insomnia, worry, and rumination.

**Results:**

The sample comprised five men with a mean age of 59.9 (SD 4.4) years. Four of the patients attended all six sessions, and one patient attended all but one session. Mean HADS-A scores at baseline, post-treatment, and follow-up were 9.4 (SD 3.0), 4.2 (SD 3.0), and 4.0 (SD 2.5), and for HADS-D 8.6 (SD 3.3), 3.0 (SD 3.7), and 1.6 (SD 1.5), respectively. The results showed clinically significant changes in anxiety, depression, psychiatric disorders, insomnia, worry, and rumination. Statistically significant changes were found from pre- to post-treatment scores for HADS-A and worry, which were maintained at follow-up, and HADS-D scores significantly decreased from pre-treatment to 6-months follow-up.

**Conclusions:**

ATT in a group format appears to be a feasible stand-alone metacognitive treatment for CHD patients. An adequately powered randomized controlled trial is warranted.

## Introduction

Cardiovascular disease (CVD) is the leading cause of disease burden and premature death worldwide with coronary heart disease (CHD) being its most common clinical presentation (Murray and Lopez, 1997; Smaardijk et al., 2019). As the short-term mortality rate of myocardial infarction (MI) has decreased during the past decades due to widespread use of coronary interventions and effective medical treatment, more CHD patients survive and are in need for effective secondary prevention strategies (Visseren et al., 2021).

Significant symptoms of depression and anxiety are common (30–40%) in CHD patients and are associated with an up to 2.0 times increased risk of cardiovascular events and mortality (van Melle et al., 2004; Watkins et al., 2013; Smaardijk et al., 2019), as well as with lower adherence to treatment, higher prevalence of cardiovascular risk factors (e.g., smoking), lower quality of life, increased risk of future emotional distress and higher healthcare cost compared to those without such symptoms (Wells et al., 2020). Symptoms of depression and anxiety commonly co-occur (Kessler et al., 2005) and a particular high-risk group in terms of poor CV prognosis are those CHD patients who score >8 for symptoms of both HADS-anxiety and depression (Watkins et al., 2013). Most of these previous studies have assessed anxiety and depression within weeks after the event. However, using the Hospital Anxiety and Depression Rating Scale (HADS) in the Norwegian Coronary Prevention (NOR-COR) study the prevalence estimates of significant symptoms of anxiety and depression were 21 and 13%, respectively, 2–36 months after a CHD event (Sverre et al., 2021). Moreover, longitudinal studies on the persistence of symptoms of anxiety and depression in CHD patients have shown stability or increase in symptoms over time (Konrad et al., 2016; Palacios et al., 2016; Sverre et al., 2021), which suggests that in the absence of effective treatment these symptoms in CHD patients are unlikely to naturally improve. Therefore, there is a need to identify and effectively treat depression and anxiety in these patients in order to alleviate the suffering and to improve prognosis and quality of life as well as to reduce health care cost.

A recent Cochrane review on psychological interventions for CHD patients with anxiety and depression (Richards et al., 2018) concluded that available drug and psychological treatments have low evidence quality and have only small effects on symptoms with reduction in anxiety with pooled standardized mean difference (SMD) of 0.24 and 0.27 for depression. Furthermore, small effects for quality of life, and no effects on cardiac prognosis were found (Richards et al., 2018). In addition, a systematic review of psychological interventions for CHD patients delivered by health professionals without the requirement of specific training reported no effect on anxiety and depression (Reid et al., 2013). In both reviews it was concluded that it is essential to develop and test interventions that improve both cardiac and mental health problems (Reid et al., 2013; Richards et al., 2018). The UK's NICE guidelines (CG91) on the treatment or management of depression in adults with a chronic physical health problem favor the use of psychological interventions as first-line interventions in patients with minor or mild to moderate depression due to the adverse effects of antidepressants and the resulting poor risk-benefit ratio (National Institute for Health Care Excellence, 2009). The evidence for more specific recommendations is scarce. Given the lack of effective interventions to date, there is a clear need to develop feasible and more effective psychological treatments for anxiety and depression in CHD patients.

ATT is a component of metacognitive therapy based on a transdiagnostic model of emotional disorders by Wells (2009). The self-regulatory executive function (S-REF) model provides the theoretical basis for the metacognitive model and therapy more broadly (Wells and Matthews, 1996; Wells, 2009). The S-REF model proposes that sustained inflexible styles of thinking in response to negative thoughts, feelings and beliefs lead to long-term emotional distress. An individual may for example experience the thought “I will get a new heart attack soon” and experience negative emotions (e.g., anxiety) because of the thought. If the individual easily dismisses this thought, the negative emotion associated with it is likely to dissipate. However, if the person responds with perseverative thinking (worry, rumination), the person is likely to experience persistent negative emotions. Furthermore, if the person has the belief that the sustained processing style is helpful (“If I worry about having a heart attack it is less likely to happen”) it may lead to persistence of the processing in terms of worry and rumination. Such processing of thoughts, threats, and emotions is representative of the cognitive attentional syndrome (CAS) a concept central of the S-REF model and metacognitive therapy. A core feature of the CAS is self-focused attention (SFA) that refers to the sustained, internal, and rigid focus on negative thought at the expense of flexibly engaging in the present moment. ATT aims to disrupt prolonged inflexible self-focused worry and rumination-based thinking styles that develop and maintain psychological distress. ATT seeks to weaken internal focus of attention and strengthen external focus of attention and thereby reducing preservative thinking in terms of worry and rumination. It has been suggested that the limited effectiveness of psychological interventions in CHD patients may be due to previous treatments that have not addressed the key factors linking depression to cardiac outcomes such as rumination and worry. These factors are targeted in metacognitive therapy and several intervention studies conducted in people with anxiety and depressive disorders have reported a significant reduction in rumination that is proposed as the central mechanisms for change in depressive symptoms (Kowalski et al., 2020). Type D personality and insomnia are also strongly associated with symptoms of anxiety and depression in CHD patients (Coryell et al., 2013; Frøjd et al., 2021). Moreover, insomnia is associated with rumination and worry (Frøjd et al., 2021) and both insomnia and Type D personality are independently associated with poor cardiovascular prognosis (Condén and Rosenblad, 2016; Frøjd et al., 2022; Raykh et al., 2022). Therefore, it is also relevant to assess rumination, worry, insomnia, and Type D personality as secondary outcomes that may be potentially changed by ATT.

A recent review of studies on the effectiveness of ATT (Fergus and Bardeen, 2016) found therapeutic benefits of this technique across a broad range of symptoms and disorders (panic disorder, social anxiety, depression, stressful life events, and health anxiety). A systematic review of the efficacy of ATT in non-clinical and clinical samples concluded that preliminary results suggest that ATT may be effective in treating anxiety and depressive disorders (Knowles et al., 2016). Rochat et al. (2018) concluded that MCT has a large effect on depression, anxiety and other psychological symptoms with effect sizes similar to that being reported by ATT used as a stand-alone treatment. Thus, they stated that ATT as a stand-alone intervention may be considered sufficient to challenge the CAS without requiring a more comprehensive metacognitive therapy package (Rochat et al., 2018). In line with this, it has been recommended that future studies should evaluate ATT as a true stand-alone intervention in randomized controlled trials (RCT) (Fergus and Bardeen, 2016). To date, we are only aware of two RCT studies evaluating the effectiveness of ATT in groups. One conducted in stressed students reported a significant decrease in stress (Myhr et al., 2019) and another study among students reported a significant decrease in symptoms of anxiety and depression (Haukaas et al., 2018).

Recently, a study of MCT in CHD patients showed promising effects (SMD 0.44 for HADS-anxiety and 0.47 for HADS-depression), however, ATT was not included (Wells et al., 2021). To date, there is therefore no study on ATT in CHD patients even though this treatment potentially has advantages for use in these patients. ATT treatment is brief (six to ten sessions typically) and, therefore, potentially cost-effective, which holds considerable appeal for use in increasingly busy cardiac follow-up clinics.

Since the benefits of ATT for CHD patients are unknown, although preliminary evidence is encouraging, guidance regarding the development and evaluation of ATT as a stand-alone treatment in patients without CHD suggests that it is appropriate to conduct a feasibility or exploratory study (Lancaster et al., 2004). This will inform the design of subsequent definitive trials and provide information regarding acceptability and feasibility of ATT. The present exploratory study, therefore, aimed to conduct a preliminary examination of the feasibility (recruitment, adherence with group treatment, and ATT practice), and preliminary improvements in outcomes (anxiety, depression, diagnoses, insomnia and personality) of ATT in CHD patients with significant symptoms of anxiety and depression.

## Method

### Trial Design

This was an open group-based intervention study designed to assess feasibility (proportion of eligible patients who consented to participate, attendance of group sessions and completion of ATT exercises) and usefulness of ATT, and to explore changes in anxiety and depression symptoms as well as in psychological factors known to be important for the prognosis in CHD patients in those experiencing distressing symptoms of anxiety and/or depression. The study was reported according to the CONSORT guidelines (Dwan et al., 2019). The study complies with the Declaration of Helsinki and was approved by the Regional Committee for Medical and Health research ethics (REK 2018/514). All patients gave written informed consent.

### Participants

Consecutive patients hospitalized with a CHD event at the department of cardiology at Drammen hospital 2–5 years earlier were assessed for eligibility. Inclusion and exclusion criteria for the trial were as follows: CHD is the primary somatic problem; The conditions had to fulfill the criteria of ICD-10: 125.1 [Atherosclerotic cardiovascular disease (ASCVD), 121.9 acute MI, unspecified or 122.9 (MI)]; Score >8 on either HADS-anxiety or depression subscale [associated with poor prognosis and recommended as cut-off value for clinical symptoms, with optimal sensitivity and specificity for identifying caseness (Bjelland et al., 2002), applied in similar studies for comparison of results (Wells et al., 2021)]; Age 18–65 years; Being able to understand and write the Norwegian language and signed written consent. Exclusion criteria were: Psychosis or cognitive impairment (screened during psychiatric assessments); Serious comorbid somatic disorders that will affect prognosis as bad or worse than CAD [all cancer including malignant melanoma, except skin cancer, renal failure (defined as GFR <40), hepatic failure (defined as ALAT > 210)]; Chronic heart failure (LVEF <40%); Other somatic illness than CAD is experienced as main somatic problem; Current or past neurological illness, traumatic brain injury; Current alcohol and/or substance dependency disorders, psychotic disorders, bipolar disorder, developmental disorders; Concurrent psychological intervention for emotional distress or antidepressant or anxiolytic medication initiated during previous 8 weeks and acute suicidality.

### Diagnostic Assessments

In order to take into account the presence or absence of affective and anxiety disorders, all patients were assessed with the structured clinical interview for DSM-IV axis I (SCID-I) (First et al., 1997). We also assessed axis II personality disorders by using the structured clinical interview for DSM-IV axis II disorders (SCID-II). It consists of 94 questions which cover the criteria for each of the 10 personality disorders. Four or five of the items must be scored as completely fulfilled to give a diagnosis, except for antisocial personality disorder.

### Diagnostic Reliability

All diagnostic assessments were confirmed in discussions between the rater and the last author (CP) who both have extensive experience with diagnostic interviews SCID-I/P and SCID-II. When in doubt of a diagnosis, a consensus was reached. The researcher who conducted the interviews has previously obtained excellent reliability estimates in diagnostic assessments of anxiety and depression disorders (Sagen et al., 2010; Einvik et al., 2011).

### Outcomes

Anxiety and depression symptoms were assessed using the HADS (Zigmond and Snaith, 1983) and were the primary outcomes. The HADS measures symptoms of anxiety (7 items) and depression (7 items). Items are rated on a 4 point (0–3) scale with higher scores indicating higher levels of symptoms. Scores range from 0 to 21 and is categorized as normal (0–7), mild (8–10), moderate (11–14), or severe (15–21). Other outcome measures included: Type D Personality assessed by the DS-14 (Denollet, 2005), a 14 item scale comprising two seven item subscales, negative affectivity and social inhibition. Each item is rated on a five-point (0–4) scale. A person is identified as having Type D personality if the scores on both subscales are ≥10. Insomnia was assessed by the Bergen Insomnia Scale (BIS: Pallesen et al., 2008) which is a six-item questionnaire based on the criteria for the clinical diagnosis of insomnia described in the Diagnostic and Statistical Manual of Mental Disorders, fourth version (DSM-IV-TR). Those who fulfilled these criteria according to the scoring criteria described by Pallesen et al. were categorized with “insomnia.” Rumination was measured by the Ruminative Response Scale (RRS; Nolen-Hoeksema and Morrow, 1991), a 22-item questionnaire. Each item is rated on a four-point (1 to 4) scale. Total scores range from 22 to 88, with higher scores indicating higher levels of rumination. Worry was assessed by the Penn State Worry questionnaire (PSWQ; Meyer et al., 1990), with 16 items, each rated on a five-point (1–5) scale. Scores range from 16 to 80, higher scores indicate a greater predisposition to worry. We used validated Norwegian versions of these questionnaires (Pallesen et al., 2006; Bergvik et al., 2010; Leiknes et al., 2016). Furthermore, a reliability study conducted in the sample from which the patients in our study were recruited showed adequate reliability scores (Peersen et al., 2017).

### Procedure

Consecutive patients were contacted by phone for a preliminary screening with the HADS and if eligible, they were offered an appointment at the cardiac department for further evaluation. If patients met the inclusion criteria, they were informed about the study and requested to sign an informed consent to participate in the study.

### Intervention

ATT was developed by Wells (1990, 2009) and it requires individuals to learn to direct and control their attention flexibly. The protocol has been studied extensively in anxiety disorders and in depression (Papageorgiou and Wells, 2000). The intervention followed the protocol as described by Wells (2009). The participants were first introduced to the rationale for treatment and explanation of internal and external focus of attention. A generic rationale was provided (Wells, 2009, p. 59–60) detailing how ATT may aid in controlling rigid and biased focus of attention and subsequently leading to improvements in psychological distress. It was emphasized that ATT was not intended to use for coping with distress.

ATT consisted of 6 weekly group sessions of 60–90 min duration. The first session lasted for 90 min and consisted of group ground rules, psychoeducation of the nature of symptoms of anxiety and depression in CHD patients, nature of and rationale for ATT as a method of counteracting excessive self-focus. The ATT rationale included a “healing metaphor” in which the persistence of symptoms was related to overthinking (Wells, 2009, p. 138). Participants were informed that they can learn to control worry and rumination (overthinking) with the help of ATT. As part of the ATT protocol, a self-attention rating was completed in response to the item “At this moment in time how much is your attention focused on your external environment” scored on a scale ranging from −3 (entirely externally focused) to + 3 (entirely self-focused) before and after the practice of ATT.

ATT practice involved an auditory attention task that required 12 min and was provided to participants as CD or mp3 file and practiced during each session and in-between sessions for homework. ATT instructs the patients to focus on external sounds and spatial attention in fixed sequences of first, selective attention, then rapidly changes of the attention and finally divided attention. The participants were encouraged to share their experience during ATT in the session and to ask questions. After introduction of ATT and practice in session, the patients were asked to carry out ATT twice daily as a homework assignment and a brief in-session practice of ATT was implemented throughout the treatment. Compliance and response to ATT were recorded for each patient.

In order to maintain treatment fidelity and adherence, each session was discussed thoroughly with one of the authors (CP) with considerable experience and competence in ATT. Furthermore, the therapist (TD) who performed the treatment has published studies on group metacognitive therapy in depressed patients (Dammen et al., 2014, 2016) and thus was familiar with ATT treatment. In line with the treatment manual (Wells, 2009), the structure of the sessions was as follows: Review of last session and homework assignment, ATT practice with scoring of self-focus of attention before and after, homework with ATT practice.

### Data analysis

Non-parametric analyses using Wilcoxon's signed ranks test were used for skewed data. Tests of significance were 2-tailed, but no correction was made for multiple comparisons given that this is a feasibility study in which we are less concerned about type 1 error. Treatment effect sizes for changes in symptom scores between pre- and post-treatment and pre- treatment and follow-up were calculated using Cohen's *d* statistic, which was calculated as M_1_ M_2_/SD_pooled_, where 0.2 indicates small, 0.5 medium, and 0.8 large effects (Cohen, 1988). Missing data were replaced by carrying the last observation forward (LOCF); although this assumption of stability is likely to bias results when comparing treatments (Hamer and Simpson, 2009), it is arguably a conservative assumption in an uncontrolled open trial such as this study.

## Results

**Figure 1:**
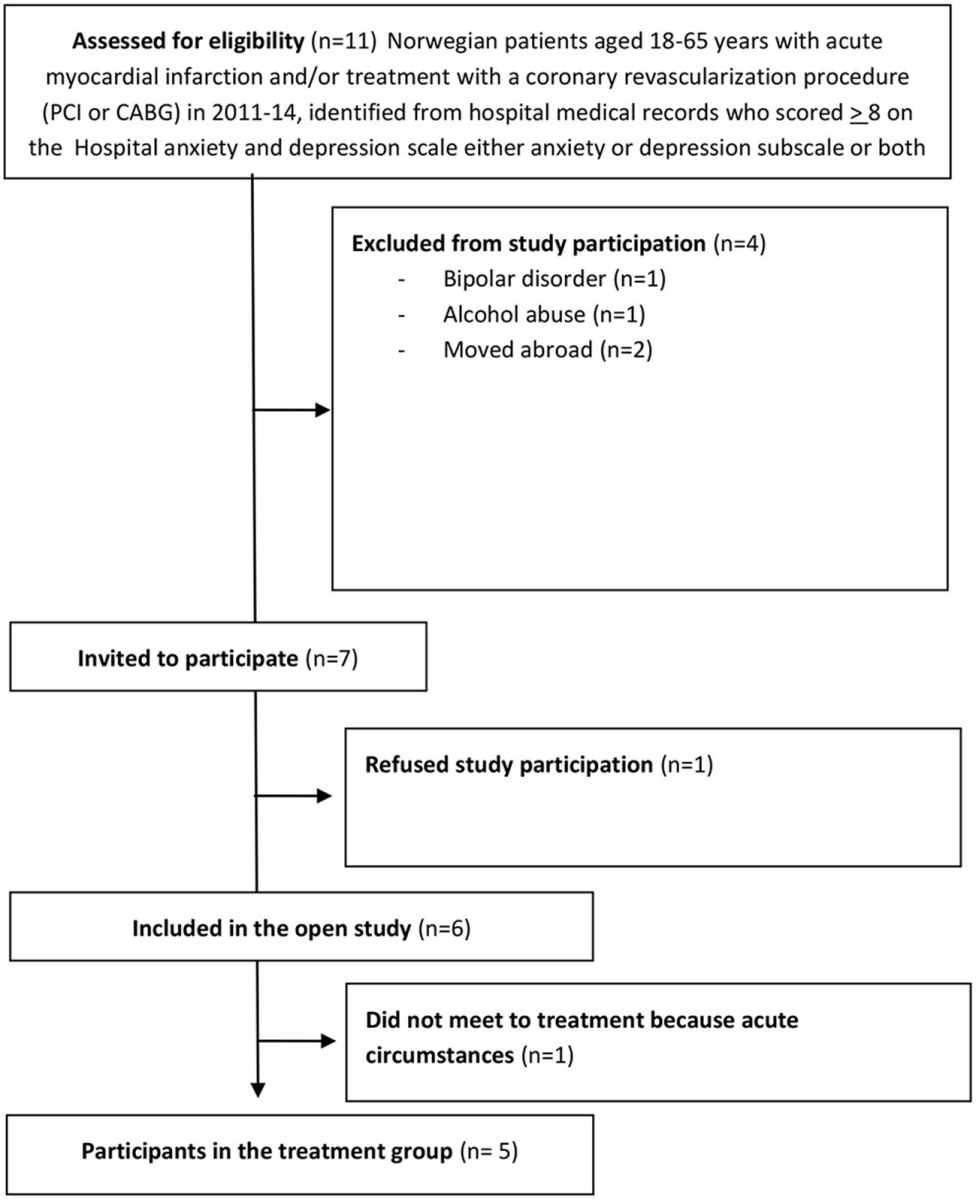
Study flow chart.

The characteristics of the sample are presented in Table 1. The CONSORT diagram for the study is provided in Figure 1.

**Table 1:** Patient characteristics at baseline.

Age, mean (SD)	59.9 (4.4) years
Gender, men (*n*)	5
Major depressive disorder (MDD)	2
Generalized anxiety disorder (GAD)	2
Social phobia (SP)	1
Obsessive compulsive personality disorder	1
Avoidant personality disorder	1
Dependent personality disorder	1
Type D personality	3
Insomnia	4
HADS-A, mean (SD)	9.40 (2.97)
HADS-D, mean (SD)	8.60 (3.29)
DS-14 NA, mean (SD)	14.00 (5.79)
DS-14 SI, mean (SD)	12.40 (9.29)
PSWQ, mean (SD)	52.80 (6.76)
RRS, mean (SD)	43.40 (8.85)

*SD, standard deviation; HADS-A, Hospital anxiety and depression scale–anxiety subscale; HADS-D, Hospital anxiety and depression scale–depression subscale; DS-14, Distressed type D scale; NA, negative affectivity; SI, social inhibition; PSWQ, Penn state worry questionnaire; RRS, Ruminative responses scale*.

No participants were receiving antidepressant or anxiolytic medication. All participants had a current Axis I disorder and one also had personality disorders. Four had a depressive disorder, whereas one had an anxiety disorder only (Generalized anxiety disorder). All participating patients were male and mean age was 59.9 (SD 4.4) years.

In terms of feasibility of the trial, it is clear that recruitment was relatively successful; we had a final sample of 5 participants from 7 eligible patients only 1 participants declined and one consented, but withdrew from the study due to acute circumstances before the first session. Thus, 6 of 7 (85.7%) eligible patients consented to participate suggesting good willingness to participate in the study and to consider ATT, within this population. Adherence to ATT was acceptable, with one participant not attending one session, and all receiving at least 5 sessions. No adverse events were reported. Three patients reported having conducted the ATT home assignments in total 27 times, whereas two reported 37 times (mean 31, SD 5.48).

Analyses of the effects of ATT on our outcome measures, including both total score and subscales, at pre- and end of treatment, as well as at 6 months follow up, are shown in Table 2; both tests of significance (Wilcoxon Signed Rank Test) and effect sizes (Cohen's *d*) are reported. HADS-anxiety and worry (PSWQ) symptoms were significantly reduced at the end of treatment and at follow-up. HADS-depression, negative affectivity and social inhibition were significantly reduced from pre-treatment to follow-up.

**Table 2:** Changes in anxiety, depression, negative affectivity, social inhibition, worry, and rumination.

**Wilcoxon signed ranks test**	**Baseline mean (SD)**	**End of treatment mean (SD)**	**Follow up mean (SD)**	**Pre- to post-treatment**				**Post-Treatment to follow-up**				**Pre-Treatment to follow-up**			
				**W/Z**	***p***	***d***	**95% CI**	**W/Z**	***p***	***d***	**95% CI**	**W/Z**	***p***	***d***	**95% CI**
HADS-A	9.40 (2.97)	4.20 (2.95)	4.00 (2.45)	−2.02*	0.04	1.67	0.22–3.06	−0.18*	0.85	0.10	−0.78–0.98	−2.03*	0.04	2.77	0.72–4.79
HADS-D	8.60 (3.29)	3.00 (3.74)	1.60 (1.52)	−1.83*	0.07	1.00	−0.132–2.08	−1.07*	0.29	0.56	−0.423–1.48	−2.03*	0.04	1.63	0.20–2.99
DS14-NA	14.00 (5.79)	9.80 (8.32)	9.80 (8.41)	−1.83*	0.07	1.10	−0.08–2.20	0.00**	1.00	0.00	−0.88–0.88	−1.84*	0.04	1.23	−0,01–2.39
DS14-SI	12.40 (9.29)	9.00 (8.51)	9.00 (8.06)	−1.84*	0.07	1.09	−0.09–2.19	0.00**	1.00	0.00	−0.79–0.79	−1.89*	0.06	1.74	0.26–3.17
PSWQ	52.80 (6.76)	39.40 (9.34)	39.60 (11.63)	−2.02	0.04	1.17	−0.04–2.31	−0.18***	0.85	−0.03	−0.91–0.85	−2.02*	0.04	1.20	−0.24–2.35
RRS	43.40 (8.85)	36.20 (13.54)	38.2 (14.01)	−1.21*	0.23	0.59	−0.40–1.52	0.00**	1.00	−0.32	−1.21–0.60	−0.14*	0.89	0.37	−0.56–1.26

^*^, W positive (based on positive ranks); ^**^, W equal (the sum of negative ranks equals the sum of positive ranks); ^***^, W negative (based on negative ranks).

*W/Z, Wilcoxon Signed Ranks Test; p, 2-tailed significance p-value; d, Cohen's d point estimate; SD, standard deviation; HADS-A, Hospital anxiety and depression scale–anxiety subscale; HADS-D, Hospital anxiety and depression scale–depression subscale; DS-14, Distressed type D scale; NA, negative affectivity; SI, social inhibition; PSWQ, Penn state worry questionnaire; RRS, Ruminative response scale*.

Table 3 shows the changes reported in psychiatric disorders, personality disorders, type D personality and insomnia, as measured by, respectively, SCID I, SCID II, DS14, and BIS, from pre-treatment to post-treatment and follow-up. A clinically significant reduction in the number of patients with axis I disorders as well as axis II disorders was observed. The number of patients with insomnia was reduced from four to one from pre-treatment to follow up. The type D personality status was not changed in those three who reported type D personality.

**Table 3:** Psychiatric disorders, personality disorders, type D personality, and insomnia.

	**Pre (*n*)**	**Post (*n*)**	**Follow up (*n*)**
**Psychiatric disorders**
Major depressive disorder (MDD)	2	0	0
Depression, not otherwise specified (NOS)	2	1	1
Generalized anxiety disorder (GAD)	2	0	0
Social phobia (SP)	1	0	0
**Personality disorders**
Obsessive compulsive disorder (OCD-PD)	1	1	1
Avoidant personality disorder (AVP)	1	0	0
Dependent personality disorder (DPD)	1	0	0
Type D personality	3	2	3
Insomnia	4	2	1

*n, number of participants*.

## Discussion

In this pilot intervention study, we set out to investigate the feasibility, acceptability, and preliminary effectiveness of group ATT for symptoms of anxiety and depression in CHD out-patients. Our results demonstrated that ATT was both a feasible and acceptable intervention for outpatients with CHD in a group setting. Patient recruitment was good with two thirds of eligible patients consenting to participate, none withdrew from the study and the patients attended almost all of the group ATT sessions and were able to complete their homework practice. We also found significant changes in symptoms of anxiety and depression as well as clinically significant changes in insomnia and psychiatric disorders in this first stand-alone group based ATT intervention among CHD outpatients.

The recruitment rate was better than described in a study by Wells et al. (2020), where they recruited 36% of eligible patients to a feasibility study of full metacognitive group treatment in a cardiac rehabilitation setting, and 47% eligible patients were included in the final trial (Wells et al., 2021). Our recruitment rate is also higher than those reported in previous studies of psychological intervention in CHD patients ranging from 32 to 59% (Wells et al., 2021). We may speculate that the recruitment to our study may be higher than most other studies due to the nature of the treatment, and because the patients had suffered symptoms for a longer time period after the event and thus were more motivated for treatment. However, reasons for consenting to and declining participation should be investigated in future studies.

The group session attrition rate was high and all patients stated that they appreciated being in a group in which they could meet other CHD patients with similar symptoms and share experiences, even though this was to a large extent limited to what was shared about the experience of ATT and training. However, the importance of non-specific treatment factors and the importance of interacting with CHD patients has also been described by McPhillips and colleagues in a qualitative study of MCT in CHD patients (McPhillips et al., 2021).

All patients complied with ATT homework practice exercises between group sessions. All practiced ATT session well above 13 times which has been regarded as the minimum “dose” of most psychological treatment sessions (Hansen et al., 2002) and which has been applied as a “benchmark” number of sessions in previous studies of ATT (Fergus and Hiraoka, 2018). However, the minimum number of ATT sessions required for optimal effectiveness remains unknown. Single case studies suggest an average of six ATT sessions to be sufficient for 6–12 months long-term benefit (Papageorgiou and Wells, 2000; Knowles et al., 2016). However, we did not find significant changes from pre-to mid-term treatment on any outcome measure (data not shown) in the total group, even one patient had a significant symptom decline. Therefore, our results indicate that six group sessions and more than 13 ATT sessions are required to obtain significant changes in symptoms of anxiety and depression for most CHD patients. Our observation that some require a limited number of session whereas other require more extensive treatment in order to obtain effect is similar to what we have observed in studies in psychiatric settings. Future studies of larger sample size may assess the outcome after each session and various number of ATT practice in order to identify the number of sessions necessary for effectiveness as well as predictors to identify those who are in need of fewer or more ATT sessions. Furthermore, we noticed, as in previous studies (Wells et al., 2021) that some misinterpreted the intention of ATT as being a way of coping or help “not to think about negative thoughts.” This is regarded as distraction and thought suppression and might strengthen the CAS instead of removing it.

In the last session, the patients reported that what they had learned from therapy was where to have their focus of attention, to let negative thoughts be without giving attention to them, and to choose their focus of attention on external rather than internal events. However, one participant stated he had learned not to focus on negative thoughts. Therefore, there is a need to correct misunderstandings and foster the understanding of the rationale for ATT. However, the rationale seemed to be understood by all apart from one patient.

Our study demonstrated that group ATT was associated with significant reductions in symptoms of anxiety and worry at both end of treatment and at follow up. Depression symptoms as well as negative affectivity were also significantly reduced from pre-treatment to follow up. Our effect sizes indicate that the magnitude of statistical change associated with treatment can be considered moderate to good and are higher than those previously reported for psychological treatments or full MCT group treatment in CHD patients. The reduction in negative affectivity, one of the type D personality traits, is interesting because effective treatment of type D personality is yet not known. The effect on negative effectivity should therefore also be studied in future studies. This is important because some studies suggest that particularly negative affectivity may be related to poor prognosis and further suggest a negative impact of type D personality on cardiovascular prognosis in CHD patients (Raykh et al., 2022). Noteworthy, however, type D status did not change as a consequence, but this outcome is worthy exploring in future studies. Although an exploratory study, these results clearly need to be evaluated further in an adequately powered randomized, controlled blinded trial since the effect sizes are likely to be inflated by the lack of a control condition and blinding. Importantly, however, these pilot results are in line with what has been reported in most studies summarized in a review of ATT in non-clinical samples and clinical samples in psychiatric settings with group effect sizes (0.95–1.78) in most studies (Knowles et al., 2016).

We also demonstrated that ATT is associated with clinically significant reductions in psychiatric diagnosis, as we observed a lower number of patients with both axis I disorders (MDD, GAD, Depression NOS, SP) and axis II disorders (AVP, DPD). This is in line with what has been reported in studies in psychiatric patients (Dammen et al., 2014; Hjemdal et al., 2017). A reduction in the number of patients suffering from insomnia was also observed. The results suggest that these outcomes should be further explored in a larger methodologically sound RCT.

Finally, our study suggests that ATT, a component based on a specific metacognitive model is capable of changing perseverative thinking, such as worry. Thus, there is preliminary evidence of ATT having a significant effect on depression and anxiety symptoms, and also on psychiatric diagnoses. A larger sample size would clearly be required to examine the effects of ATT in CHD patients. Given our promising clinical effects and the fact that it is a brief treatment that focuses on process rather than content of thinking, we hypothesize that ATT could be a treatment option for anxious and depressed CHD patients.

Inevitably, in a phase I exploratory trial of this kind, there are significant methodological limitations. The small sample size, which was a convenience sample, reduces statistical power, Nonetheless, we found significant effects on several outcome measures, and our primary purpose was to demonstrate feasibility, which we achieved. The sample was diagnostically heterogeneous regarding the presence of axis I and axis II disorders whereas the symptom levels of HADS-anxiety and depression were more homogenous within the range categorized as mild-moderate (Stern, 2014). Even though our baseline scores were in the same category as those reported in the study by Wells et al. (2021), we cannot generalize effects to all severity levels regarding these symptoms. Furthermore, since all participants were male, we cannot generalize our results to women with CHD. Treatment fidelity was not formally assessed, but the supervision and training of the therapist who carried out the intervention was by a qualified and competent MCT supervisor. Moreover, the therapist has previously published studies of MCT including ATT and the use of a treatment manual should have improved the consistency with which ATT was delivered. The assessor was not blind to the presence of treatment and the trial was uncontrolled which affords no protection from rater bias and cannot control for factors such as time and non-specific treatment effects. Therefore, it is likely that the effect sizes observed are inflated. However, all participants reported significant distress longer time up to years after the previous cardiac event which may indicate chronic distress. Finally, we did not assess psychological factors that may aid in elucidating the mechanisms for the effectiveness of treatment. Thus, the assessment of metacognitions and CAS should be included in future studies. State and trait self-focused attention should also be assessed. We used the SCID for DSM-IV criteria because this has been applied from the start of NORCOR study. Therefore, we do not have diagnostic assessment according to the more recent DSM-V criteria.

To overcome these limitations, a randomized controlled trial is now needed to evaluate the clinical and health-economic effects of ATT for CHD patients with significant anxiety and depression symptoms in comparison with wait list control. The HADS would seem to be an outcome that is sensitive to change, easy to administer and would allow comparison with other treatment trials in this field (Wells et al., 2021).

In conclusion, ATT in a group format appears to be a feasible stand-alone metacognitive treatment for CHD patients with significant symptoms of anxiety or depression. An adequately powered randomized controlled trial is now warranted.

## Data Availability Statement

The datasets presented in this article are not readily available because according to Norwegian legislation, the Norwegian Data Protection Authority, and the Committee of Ethics, we are not allowed to share original study data publicly. However, the essential generated data are available from the corresponding author on reasonable request. Requests to access the datasets should be directed to toril.dammen@medisin.uio.no.

## Ethics Statement

The studies involving human participants were reviewed and approved by Regional Committees for Medical Research Ethics South East Norway. The patients/participants provided their written informed consent to participate in this study.

## Author Contributions

TD and CP contributed to the idea and design of the study. TD contributed to the data collection and scoring. TD and KT contributed to the data analysis, interpretation, and responsible for the first draft of the manuscript. All authors contributed significantly to the final version of the manuscript.

## Funding

The study was funded by grants from South-Eastern Norway Regional Health Authority (Helse Sør-Øst RHF/Grant No. 2019125) and the University of Oslo. Open Access publication was funded by the University of Oslo.

## Conflict of Interest

The authors declare that the research was conducted in the absence of any commercial or financial relationships that could be construed as a potential conflict of interest.

## Publisher's Note

All claims expressed in this article are solely those of the authors and do not necessarily represent those of their affiliated organizations, or those of the publisher, the editors and the reviewers. Any product that may be evaluated in this article, or claim that may be made by its manufacturer, is not guaranteed or endorsed by the publisher.
